# Cystoid macular edema and visual loss as sequelae to interferon alpha treatment of systemic hepatitis C

**DOI:** 10.4103/0301-4738.60088

**Published:** 2010

**Authors:** Hiten G Sheth, Michel Michaelides, Dilani Siriwardena

**Affiliations:** Moorfields Eye Hospital, City Road, London EC1V 2PD, UK

**Keywords:** Hepatitis C, interferon alpha, cystoid macular edema, visual loss

## Abstract

Hepatitis C virus infection and interferon treatment may be associated with retinopathy but visual function is generally unaffected. This paper reports the rare occurrence of unilateral macular edema with visual loss. We present an interventional case report with fundus photograph and optical coherence tomography (OCT). A 48-year-old white male with hepatitis C, treated with a six-month course of pegylated interferon alpha and ribavirin, complained of gradual reduction in the vision of his left eye. Visual acuities were 20/16 right and 20/400 left with clinical examination and OCT confirming cystoid macular edema.

This report shows that cystoid macular edema may rarely occur in association with hepatitis C infection and/or interferon therapy. Physicians and ophthalmologists should be alert to this potential but infrequent association as the resultant visual loss is a significant potential complication that should be discussed when obtaining informed consent for interferon treatment.

Hepatitis C, virus (HCV) may be associated with an ischemic retinopathy thought to be secondary to HCV-induced vasculitis.[1] Contemporary treatment for chronic systemic HCV infection comprises interferon alpha in combination with ribavirin. The side-effects of interferon include a flu-like illness, fever, fatigue, nausea, hair loss and depression. Interferon treatment has been associated with a retinopathy, characterized by cotton wool spots and retinal hemorrhages, in several prospective case series and has also been reported to cause Vogt-Koyanagi-Harada disease.[2–6] Despite this, patients rarely report subjective visual problems and visual function is generally maintained.

Here we present optical coherence tomography (OCT) evidence of cystoid macular edema (CME) probably due to pegylated interferon alpha treatment for hepatitis C in an otherwise healthy individual with none of the common risk factors for CME. To our knowledge, only two similar cases of this rare and emerging phenomenon have previously been reported worldwide.[7,8]

## Case Report

**Figure 1:**
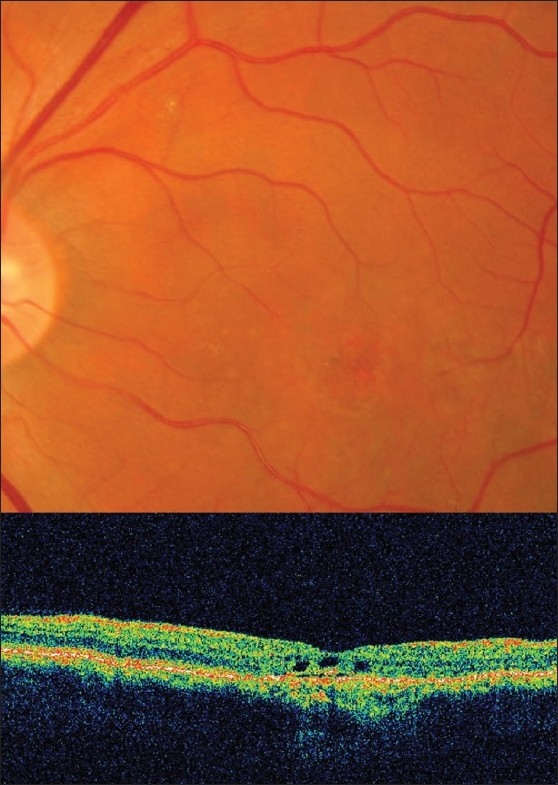
Fundus photographs and optical coherence tomography scans at first presentation showing cystoid macular edema of the left eye

A 48-year-old white male was referred to the ophthalmic accident and emergency by his general practitioner. He gave a six-month history of gradual, painless loss of central vision in his left eye. Visual acuities were 20/16 in the right eye unaided and 20/400 best corrected in the left eye. Both eyes were white with no intraocular inflammation and left eye CME was confirmed on slit-lamp biomicroscopy. Furthermore, OCT showed three symmetric cystic spaces in the external plexiform layer, increased free space between the neurosensory epithelium and retinal pigmented epithelium and disorganization of internal retinal architecture at the macula [Fig. 1]-consistent with the clinical diagnosis of CME.

He was known to be a chronic carrier of HCV (diagnosed in 2004 and attributed to an episode of tattooing) and had undergone a standardized six-month treatment regimen of pegylated interferon-alpha-2a (Pegasys 18 mcg) subcutaneous injections and oral ribavirin 600 mg twice daily under the gastroenterology service of a neighboring hospital. The visual loss had started some weeks into his course, with no subjective improvement on completing the full six-month course, although his viremia was successfully cleared. He had no previous ophthalmic history such as trauma or operations and the left eye was not amblyopic. He was on no other medication and there was no other medical history, including diabetes or hypertension. He was a non-smoker.

Topical treatment was commenced in the form of ketorolac 0.5% eye drops four times daily and dexamethasone 0.1% eye drops four times daily to the left eye. At the three-month follow-up he reported no subjective improvement in vision and acuity remained poor at 20/200 despite a repeat OCT scan showing resolution of edema. Fundus fluorescein angiogram and fundus autofluorescence confirmed macular retinal pigment epithelial atrophy. Active management and further follow-up were declined by the patient.

## Discussion

The incidence of retinopathy associated with interferon treatment varies in literature from 18-86%.[1] The most common lesions seen are cotton wool spots and retinal hemorrhages and actual subjective visual complaints or documented visual loss is infrequent. Our case is highly unusual in both the pathology and also in terms of the level of visual loss. Such ocular side-effects or associations are extremely rare and whilst routine ophthalmic screening at baseline and through the course of peg-IFN-alpha and ribavirin therapy may not be indicated, this case demonstrates that early ophthalmic assessment and treatment in the event of subjective decrease in vision may improve the final visual prognosis.

There are only two similar reports published.[7,8] The first case was of a 24-year-old male who developed bilateral CME associated with florid retinopathy, the second was a 37-year-old male who developed macular edema with hemorrhages and cotton wool spots. Concurrent hypoalbuminemia and thrombocytopenia was reported in both these patients and cessation of interferon therapy was associated with improvement of vision and normalization of blood parameters. Our patient differs in that the pathology was confined to the peri-foveal area and the visual deterioration was monocular and persistent.

Hypertension and diabetes mellitus have been shown to be risk factors in the development or progression of interferon-associated retinopathy in HCV-infected individuals. However, neither was identified in our patient.

In summary, we present a rare case of CME with attendant visual loss which may be causally related to interferon alpha treatment of hepatitis C. Ophthalmologists and physicians should be aware of the possible ocular side-effects of interferon and whilst treatment of the systemic disease must take priority, we suggest that the potential for transient or longer-term visual loss be discussed when obtaining informed consent for interferon therapy.
